# LONG-TERM POSITIVE AND NEGATIVE CONSEQUENCES OF THE COVID-19 PANDEMIC AMONG OLDER ADULTS

**DOI:** 10.1093/geroni/igac059.1961

**Published:** 2022-12-20

**Authors:** Carolyn Aldwin, Soyoung Choun, Maria Kurth, Hye Soo Lee, Dylan Lee, Heidi Igarashi

**Affiliations:** Oregon State University, Corvallis, Oregon, United States; Oregon State University, Corvallis, Oregon, United States; Oregon State University, Corvallis, Oregon, United States; Oregon State University, Corvallis, Oregon, United States; Touro University Nevada, Henderson, Nevada, United States; Oregon State University, Corvallis, Oregon, United States

## Abstract

Despite their greater physiological vulnerability, community-residing older adults have shown surprising psychological resilience, at least at the beginning of the COVID-19 pandemic. However, a handful of reports suggest that older adults’ well-being has decreased after a few months, although others have suggested a recovery after a year (Schlomann et al., 2021). The purpose of this study was to examine change in change from baseline (April-May, 2020) to a 13- month follow-up (June, 2021). We analyzed data from 162 older adults with complete data at both time points. Mean age at baseline was 72, SD = 7.6, range = 51-96; 71% were female, 13% were minorities; 74% were married, 71% retired, and most (85%) had at least a BA. Linear modelling showed that there were only marginal increases in the number of problems across time (B = .25, p = .08), but their severity did not increase. There were no significant changes in depression, anxiety, loneliness or physical symptoms over this time period. The modest increase in problems may have been offset by an end to being in lockdown and an increase in social contacts which doubled over this time period, B = 1.65, p <.001). More troubling was that self-reported resilience decreased, B = -.92, p < .01), as did the ability to perceive positives in this situation, B = -2.46, p < .01), and self-reported cognitive problems increased, B=.67, p < .01). Thus, the results showed decidedly mixed effects, suggesting individual differences in long-term adaptation to COVID.

